# Molecular Docking as a Therapeutic Approach for Targeting Cancer Stem Cell Metabolic Processes

**DOI:** 10.3389/fphar.2022.768556

**Published:** 2022-02-21

**Authors:** Babak Arjmand, Shayesteh Kokabi Hamidpour, Sepideh Alavi-Moghadam, Hanieh Yavari, Ainaz Shahbazbadr, Mostafa Rezaei Tavirani, Kambiz Gilany, Bagher Larijani

**Affiliations:** ^1^ Cell Therapy and Regenerative Medicine Research Center, Endocrinology and Metabolism Molecular-Cellular Sciences Institute, Tehran University of Medical Sciences, Tehran, Iran; ^2^ Proteomics Research Center, Shahid Beheshti University of Medical Sciences, Tehran, Iran; ^3^ Integrative Oncology Department, Breast Cancer Research Center, Motamed Cancer Institute, ACECR, Tehran, Iran; ^4^ Reproductive Immunology Research Center, Avicenna Research Institute, ACECR, Tehran, Iran; ^5^ Endocrinology and Metabolism Research Center, Endocrinology and Metabolism Clinical Sciences Institute, Tehran University of Medical Sciences, Tehran, Iran

**Keywords:** cancer, cancer stem cells, drug designing, metabolic processes, molecular docking

## Abstract

Cancer stem cells (CSCs) are subpopulation of cells which have been demonstrated in a variety of cancer models and involved in cancer initiation, progression, and development. Indeed, CSCs which seem to form a small percentage of tumor cells, display resembling characteristics to natural stem cells such as self-renewal, survival, differentiation, proliferation, and quiescence. Moreover, they have some characteristics that eventually can demonstrate the heterogeneity of cancer cells and tumor progression. On the other hand, another aspect of CSCs that has been recognized as a central concern facing cancer patients is resistance to mainstays of cancer treatment such as chemotherapy and radiation. Owing to these details and the stated stemness capabilities, these immature progenitors of cancerous cells can constantly persist after different therapies and cause tumor regrowth or metastasis. Further, in both normal development and malignancy, cellular metabolism and stemness are intricately linked and CSCs dominant metabolic phenotype changes across tumor entities, patients, and tumor subclones. Hence, CSCs can be determined as one of the factors that correlate to the failure of common therapeutic approaches in cancer treatment. In this context, researchers are searching out new alternative or complementary therapies such as targeted methods to fight against cancer. Molecular docking is one of the computational modeling methods that has a new promise in cancer cell targeting through drug designing and discovering programs. In a simple definition, molecular docking methods are used to determine the metabolic interaction between two molecules and find the best orientation of a ligand to its molecular target with minimal free energy in the formation of a stable complex. As a comprehensive approach, this computational drug design method can be thought more cost-effective and time-saving compare to other conventional methods in cancer treatment. In addition, increasing productivity and quality in pharmaceutical research can be another advantage of this molecular modeling method. Therefore, in recent years, it can be concluded that molecular docking can be considered as one of the novel strategies at the forefront of the cancer battle via targeting cancer stem cell metabolic processes.

## Graphical Abstract

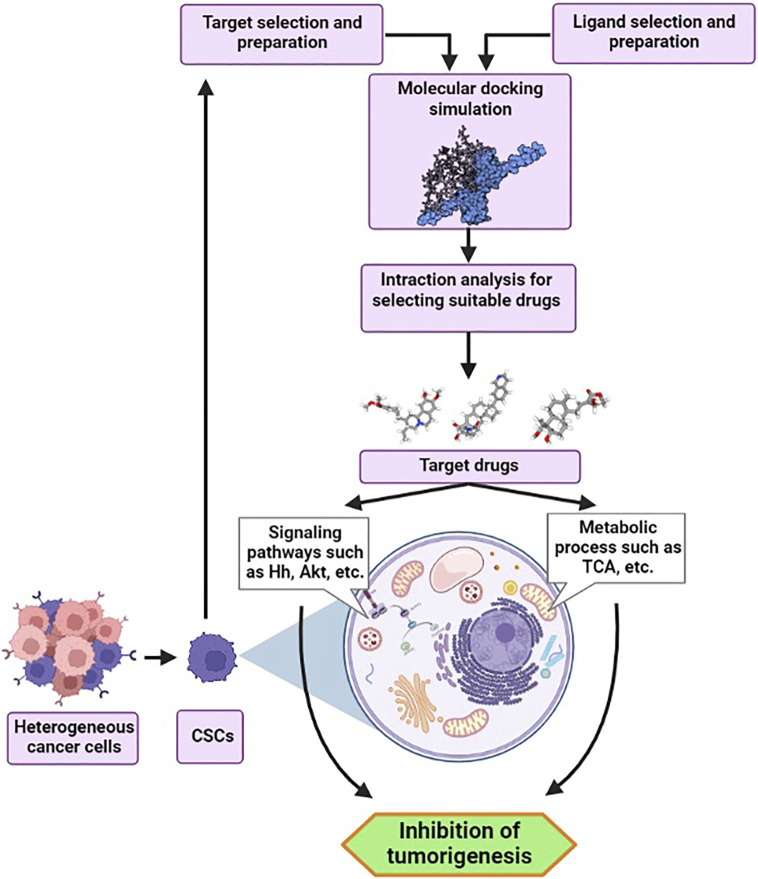

## Introduction

Cancer is considered as one of the worldwide life-threatening and the leading causes of human mortality (Vineis and Wild, 2014; Organization, 2020). According to the latest data released by the International Agency for Research on Cancer (IARC) on 14 December 2020, the annual incidence of cancer in 2020 reached 19.3 million cases and 10 million deaths. Furthermore, evidence based on the World Health Organization (WHO) suggests that there would be 29.5 million new cancer diagnoses and 16.4 million cancer deaths per year by 2040 (Shah et al., 2019; Sung et al., 2021). Accordingly, given the rapid development of oncology researches and the advancement of novel biotechnology approaches, determining different aspects of cancer progression can pave the way for improved cancer prognosis and treatment alternatives (Goyal et al., 2006; Charmsaz et al., 2018; Pucci et al., 2019). Herein, one of the challenges in the field of cancer treatment is the heterogeneity of tumor cells, which may lead to anti-cancer drug resistance or cancer treatment failure. Therefore, a full understanding of tumor heterogeneity can provide a clear picture of cancer progression and lead to the discovery of new cancer therapy options by researchers (y Cajal et al., 2020). Tumor heterogeneity is a condition in which tumor cells differ in various biological aspects such as function, differentiation, tumorigenesis, and sensitivity to anti-cancer therapies (Prager et al., 2019). In addition, depending on the type of heterogeneity, heterogeneous groups of tumor cells can have the same or distinct genomic content (Prager et al., 2019). In addition, heterogeneous populations of tumor cells can have the same or different genome content depending on the type of heterogeneity (Bedard et al., 2013). Hereupon, tumor heterogeneity can be divided into three types: 1) intertumor heterogeneity which is related to the variation of tumor cells among different patients, 2) intersite heterogeneity which is referred to the variation of cells among distinct tumors within a patient such as tumors in the primary site and tumors in the metastatic site, and 3) intratumor heterogeneity which is linked with heterogeneous populations of cells in a single tumor (Piraino et al., 2019). Oncology studies were shown that the cancer stem cells (CSCs) model is one of the models responsible for the generation of heterogeneous populations of cells, especially intratumor heterogeneity type (Prasetyanti and Medema, 2017; Turnquist et al.). Moreover, it can be caused by different factors such as genetics, epigenetics, and various micro-environmental features (Wang et al., 2015). Indeed, CSCs are a subgroup of cancerous tumor cells that display stemness abilities in the same manner as normal stem cells. For instance, they can self-renew to form the same daughter cells and give rise to differentiated multiple lineages of cells which form tumors. Additionally, the quiescence state is one of the distinguishing characteristics of cancer and normal stem cells, and it can play a role in therapeutic resistance and cancer progression (Hung et al., 2019; Lee et al., 2020). Furthermore, CSCs can make the treatment process more challenging because of their resistance to therapeutic approaches such as chemo and radiation therapies. The mentioned therapeutic resistance can be due to a variety of factors and mechanisms, including tumor environment, epigenetic effects, multidrug resistance proteins (MRPs) expression, various signaling pathways, effective mechanisms in DNA damage resistance, and the epithelial-to-mesenchymal transition (EMT) process (Phi et al., 2018). On the other hand, the function of metabolic pathways and processes are crucial in the growth, proliferation and survival of CSCs. In this respect, many investigations at the cellular and molecular level were indicated that unique forms of metabolic processes such as oxidative phosphorylation (OXPHOS), carbohydrate, and lipid metabolisms are observed in CSCs (Chae and Kim, 2018; Yadav et al., 2020). Therefore, the science of metabolomics, as well as the understanding of alterations associated with metabolic processes, could be useful in recognizing CSC behaviors and developing specific therapeutic methods for various types of cancers (Gilany et al., 2018; Rahim et al., 2018; Arjmand, 2019a, 2019b; Goodarzi et al., 2019; Larijani et al., 2019; Tayanloo-Beik et al., 2020), as well as the understanding of alterations associated with metabolic processes, could be useful in recognizing CSC behaviors and developing specific therapeutic methods for various types of cancers (Cuyàs et al., 2017). Additionally, scientists have been pushed to employ targeted approaches for treating cancer due to the problems in CSCs resistance to therapeutic methods. Molecular docking is one of the targeted approaches that play an important role in drug discovery and pharmaceutical researches. This computer-assisted drug design method is based on mathematical algorithms in which the effective biological binding-conformation between the drug and the target molecule can be evaluated. Indeed, the mentioned drug designing is based on the molecular structure that makes it possible to model and predict the molecular interactions as well as evaluate the biochemical processes (Meng et al., 2011; Phillips et al., 2018). Hereupon, in the present study, the cellular and molecular characteristics, signaling pathways, metabolic processes, and drug resistance of CSCs have been reviewed. We have also focused our discussion on molecular docking as a novel therapeutic approach in CSCs targeting.

## The Biology of Cancer Stem Cells

CSCs are a subset of cancer cells or tumor-initiating cells (TICs) that serve as stem cells and contribute to the original tumor’s phenotypic variety (Lobo et al., 2007). They are found in variable amounts in different tumors. Furthermore, evaluating cell surface markers is the main strategy for detecting CSCs. Normal stem cells and CSCs have many similar characteristics (Jin et al., 2017; Khatami et al., 2019) such as 1) Self-Renewal (Lobo et al., 2007) 2) Differentiation capacity (Mohr et al., 2015) 3) Tumorigenesis (Zhu and Fan, 2018) 4) Capacity of developing resistance to drugs/cytotoxic substances and radiation (Schöning et al., 2017). Despite their similarities, there are some distinctions between cancer and somatic stem cells. The first is the origin of these two types of stem cells: natural somatic stem cells arise during embryonic development and separate from each other. They differentiate and produce a variety of mature cells, while CSCs are differentiated from normal adult stem cells or by multiple mutations in a single cell. The second is the ability to regenerate itself: somatic stem cells regenerate more regular than CSCs, although both types of cells can regenerate themselves. Finally, the organogenesis ability of these two cells is studied: both cells have the ability to organogenesis, but CSCs produce abnormal tissue, whereas somatic stem cells’ organogenesis produces normal tissue (Gjorevski et al., 2014).

### Cancer Stem Cells Isolation Markers

Since, CSCs are a small part of a big heterogeneous cell population of human cancer, isolation and division of such small human cancer cells can be a significant step in a delicate study of various aspects of cancer. Herein, identifying CSCs markers is a key factor (Tang et al., 2007). Most of the CSCs markers originate from human embryonic stem cells (hESCs) or adult stem cells (Jin et al., 2017; Najafi et al., 2019). The expression of CSCs isolation markers varies depending on a number of factors, including cell lines, tumor histotypes, isolation methods, and survey CSCs markers in vivo or *in vitro* investigations (Tirino et al., 2013). On the one hand, CSCs markers have a beneficial therapeutic effect on several types of cancers by targeting CSCs in order to eliminate tumor recurrences (Jin et al., 2017; Najafi et al., 2019). Moreover, the majority of surface markers can be harmed by interactions between enzymes and tumor tissues, and this destruction could be regarded a disadvantage (Abbaszadegan et al., 2017). Some various CSCs markers with their unique characteristics were reviewed in Table 1.

**TABLE 1:** Most frequently applied markers for cancer stem cells isolation.

CSCs Marker	Marker type	Expression location	Function	Cancer Type	References
CD44	Surface marker	Leukocytes, Endothelial cells, Hepatocytes, Mesenchymal cells	Activation of tyrosine kinase receptors by binding to extracellular matrix, Cell migration, Distinction, Increasing the speed of tumor cells entrance into blood vessels in metastasis	Breast, prostate, lung	Abbaszadegan et al. (2017); Bao et al., (2013)
CD133	Surface marker	Embryonic epithelial stem cell, Hematopoietic stem cells	Organizer of the plasma membrane topology, Conservation of plasma membrane`s lipid structure, Development of head and neck squamous cell carcinoma	Breast, prostate, lung, head, neck	Abbaszadegan et al. (2017); Bao et al. (2013); Yu and Cirillo, (2020)
CD117	Surface marker	Mesenchymal adult stem cells, Cardiac adult stem cell, Ovary	Stem cell factor`s receptor, Drug target molecules	Ovarian	Jin et al. (2017)
CD90	Surface marker	Between normal hematopoietic stem cells and leukemic CSCs	Identifying leukemic CSCs from hematopoietic stem cell subpopulation	Leukemia	Bao et al. (2013); Kumar et al., (2016)
CD24	Surface marker	Pancreatic carcinoma	Identifying CSCs in pancreas cancer	Pancreas	Gopalan et al., (2018); Jin et al. (2017)
ALDH1	Intracellular marker	Normal stem cells, Malignant stem cells, Progenitor cells	Regulator of stem cells propagation and distinction	Breast	Ajani et al., (2015); Abbaszadegan et al., (2017)
P63	Basal cell marker	Basal regenerative cells of many epithelial tissues, Prostate, Urothelial	Prostate progression, Diagnostic factor of prostate cancer	Prostate	Grisanzio and Signoretti, (2008); Klonisch et al. (2008)

ALDH1: Aldehyde dehydrogenase isoform 1; CSCs, Cancer stem cells.

### Cancer Stem Cells Signaling Pathways

In general, signaling pathways can help to precisely regulate the biological function of both CSCs and regular stem cells. Numerous signaling pathways such as Wnt, Notch, Hh, nuclear factor-κB (NF-κB), Janus kinase/signal transducers and activators of transcription (JAK-STAT), phosphoinositide 3-kinase/AKT/mammalian target of rapamycin (PI3K/AKT/mTOR), transforming growth factor (TGF)/SMAD, and peroxisome proliferator-activated receptor (PPAR) are among the intracellular factors that make a major contribution in regulating stem cell functions. Therefore, excessive or abnormal activity and even suppression of mentioned signal transduction pathways can convert the normal stem cells into cancerous. These pathways are regulated and controlled by the function of factors such as diverse proteins, microRNAs, long noncoding RNAs, and endogenous or exogenous factors, just as they change the self-renewal, survival, proliferation, differentiation, and usually tumorigenesis of CSCs (Table2). Signaling pathways interact with one another in a large and complicated network known as “crosstalk,” which is a crucial fact. Subsequently, crosstalk between signaling pathways can influence the regulation of several phenotypic features and drug resistance in CSCs (Matsui, 2016; Yang L et al., 2020). Hereupon, a deep understanding of the signaling processes underlying CSCs can pave the way for small molecules and pharmacological inhibitors to target them (Du et al., 2019).

**TABLE 2:** CSCs signaling pathways characteristics.

Signaling Pathway	Examples of Ligands	Receptors/Co-receptors	Function in CSCs	Type of Cancer		References
Wnt	WNT1, WNT2	Members of the Frizzled, LRP5and LRP6	Self-renewal	Ductal breast carcinomas	Dong, Ying, and Shi, (2019); Niehrs, (2012); Yang L et al. (2020)	
	WNT2B, WNT3	ROR1 and ROR2	Tumorigenesis	Colorectal				
	WNT3A, WNT4	PTK7	Dedifferentiation	Papillary thyroid				
	WNT5A, WNT5B	RYK	Apoptosis regulation	Esophageal				
	WNT6, WNT7A	MUSK	Metastasis	Colorectal				
	WNT7B, WNT8A, WNT8B, WNT9A, WNT9B, WNT10A, WNT10B, WNT11, WNT16	Proteoglycan families	—	—				
Notch	Delta-like proteins (DLL1, DLL3, DLL4)	Notch1	Proliferation	Glioblastoma	Karamboulas and Ailles, (2013); Yang L et al. (2020)	
	Jagged proteins (JAG1 and JAG2)	Notch2	Cell survival	Leukemia				
	—	Notch3	Self-renewal	Ovarian				
	—	Notch4	Differentiation	Colon				
	—	—	Migration	Gastric				
	—	—	Metastasis	Breast				
	—	—	Apoptosis inhibition	Pancreatic				
	—	—	Cell fate specification	Prostate				
	—	—	Asymmetric division	Skin				
	—	—	—	Non small-cell lung				
	—	—	—	Liver				
Hh	Shh	Ptch1, and to a lesser extent, Ptch2, Cdon	Self-renewal	CML	Cochrane et al., (2015); Yang L et al. (2020)	
	Ihh	Boc	Tumor growth	AML				
	Dhh	Gas1	Differentiate into transient amplifying cells	ALL				
	—	—	Metastasis	Glioma, Multiple Myeloma				
	—	—	—	Metastatic Melanoma				
	—	—	—	Breast				
	—	—	—	Gastric				
	—	—	—	Colon				
	—	—	—	Pancreatic				
	—	—	—	Prostate				
	—	—	—	Small Cell and				
	—	—	—	Non-Small Cell				
	—	—	—	Lung Cancer				
NF-κB	Lipopolysaccharide	TLRs	Inflammation	Gastrointestinal	Yang L et al. (2020)	
	IL-1β	TNFR	Stress responses	Genitourinary				
	TNF-α	IL-1R	Cell survival	Gynecological				
	bacterial cell components	CD40	Proliferation	Head				
	—	BAFFR	Tumorigenesis	Neck				
	—	LTβR	Some key angiogenesis factors and adhesion molecules expression, Self-renewal	Breast				
	—	—	Metastasis	Multiple myeloma				
	—	—	Apoptosis regulation	Blood cancer				
JAK-STAT	ILE, PDGF-C, OSM, CXCL12, HGF, TGF-β, EGF, Gastrin, IGF, Mk, BDNF, NT-3, gp130	ILFR, PDGFR, OSMR, CXCR7, c-MET, TGFR, EGFR, GRPR, IGF1R, Notch-1/2, TrkB, TrkC, IL-6/IL-6Rα	Tumorigenesis, Metastasis, Chemoresistance, EMT transition, Proliferation, Inflammation, Survival	Prostate, Breast, Gastric, Lung	Jin, (2020)	
PI3K/AKT/mTOR	Insulin and epithelial growth factor	ErbB-1; HER1, HER2 (c-ErbB-2), HER3 (c-ErbB-3), and HER4 (c-ErbB-4) CXCR4, IGF-1R	Cell proliferation, Angiogenesis, Metabolism, Differentiation, Survival, Self-renewal, Tumorigenesis	Ovarian, Cervical, Breast, Glioblastoma, Gastric, Pancreatic, Colorectal, Prostate, Hepatocellular	Chen et al. (2019); Miricescu et al. (2021); Xia and Xu, (2015)	
TGF/SMAD	TGF-β1, 2 and 3	TGFβR1, TGFβR2	Cell proliferation, Epithelial-mesenchymal transition, Differentiation, Angiogenesis, Inflammation	Liver, Breast, Gastric, Skin, Glioblastoma, Leukemia Colorectal	Bellomo et al., (2016); Liu et al., (2018)	
PPAR	Lipid-derived substrates	PPAR-α, PPAR-δ, PPAR-γ	Proliferation, Maintenance of sphere-formation ability, Expression of CSC Markers	Colorectal, Ovarian, Glioblastoma, Breast	Kuramoto et al. (2021); Tyagi et al., (2011)	

ALL: acute lymphocytic leukemia; AML, acute myeloid leukemia; BAFFR, B cell-activating factor receptor; BDNF, Brain-derived neurotrophic factor; Boc, Brother of Cdon; CAM, cell adhesion molecule; CDON, CAM-related downregulated by oncogenes; CML, chronic myeloid leukaemia; c-Met, Mesenchymal-epithelial transition factor; CXCL, C-X-C motif chemokine ligand; CXCR, C-X-C chemokine receptor; Dhh, Desert hedgehog; DLL, Delta-like proteins; EGF, epidermal growth factor; EGFR, epidermal growth factor receptor; EMT, Epithelial-to-mesenchymal transition; ErbB-1, Erythroblastic leukemia viral oncogene homolog 1; GAS1, Growth Arrest Specific 1; Gp130, Glycoprotein 130; GRPR, Gastrin-releasing peptide receptor HER, human epidermal growth factor receptor; HGF, hepatocyte growth factor; IGF, Insulin-like growth factor; IGF1R, Insulin-like growth factor receptor 1; Ihh, Indian hedgehog; IL-1β, Interleukin 1 beta; IL-1R, Interleukin-1 receptor; IL-6, Interleukin 6; IL-6Rα, Interleukin 6 receptor alpha; ILFR, leukemia inhibitory factor receptor; JAG, jagged protein; JAK-STAT, Janus kinase/signal transducer and activator of transcription; LRP, Low-density lipoprotein receptor-related protein; LTβR, lymphotoxin beta receptor; MK, Heparin-binding growth factor Midkine; MUSK, muscle associated receptor tyrosine kinase; NF-κB, Nuclear factor kappa-light-chain-enhancer of activated B cells; NT-3, Neurotrophin-3; OSM, Oncostatin M; OSMR, Oncostatin M receptor; PDGF-C, Platelet-derived growth factor C; PDGF-R, Platelet-derived growth factor receptors; PI3K/AKT/mTOR, Phosphoinositide 3-kinase/AKT/mammalian target of rapamycin; PPAR, Peroxisome proliferator-activated receptor; Ptch, Patched; PTK7, Protein tyrosine kinase 7; ROR, Receptor tyrosine kinase-like orphan receptor; RYK, receptor tyr kinase; SHH, sonic hedgehog; TGF-β, transforming growth factor beta; TGFβR, transforming growth factor beta receptor; TLRs, Toll-like receptors; TNF-α, tumor necrosis factor alpha; TNFR, tumor necrosis factor receptor; TrkB, Tropomyosin receptor kinase B; TrkC, Tropomyosin receptor kinase C.

### Cancer Stem Cells Metabolic Processes

Metabolic reprogramming is one of critically important characteristics of CSCs compared to other cancer and non-cancer cells, which plays a pivotal role in demonstration of cell functions such as proliferation, fate determination and the cancer progression. In this process, the cellular energy metabolism used by CSCs, such as different types of hydrophobic natural compounds and organic substances metabolisms, adenosine triphosphate (ATP) production pathways, etc., differs from that of other cells. In other words, the presence of more metabolites and high-energy compounds in CSCs suggests that they have a different metabolic profile compared to the other. Furthermore, studies demonstrate that oncogenic mutations, tumor suppressants, and, particularly tumor microenvironment features, can all have a major impact on the many components engaged in such metabolic processes. Nevertheless, mentioned metabolic processes and the components involved can be considered as therapeutic targets for cancer treatment (Mukha and Dubrovska, 2020; Peixoto and Lima, 2018; Zhu et al., 2020).

#### Glycolysis

CSCs such as normal cells are used glucose through glycolysis process to gain energy and survive. The methods for glucose metabolism in a CSC include OXPHOS and glycolytic phosphorylation, which are selected based on the presence of oxygen. Moreover, they play an important role in differentiation, self-renewal and homeostasis. Generally, high glucose levels increase the number of CSCs, while low glucose levels lower the quantity of CSCs (Falahzadeh et al., 2019). CSCs are adaptable cells that can cope with a wide range of situations, including low oxygen levels, insufficient blood vessel development, hyperoxidation, and hypoxia (Luo and Wicha, 2015). If the CSCs are in a state of hypoxia (lack of oxygen), the proper metabolic pathway is chosen. Herein, they enter the glycolytic pathway, which eventually leads to the formation of lactate (Yi et al., 2018). According to Warburg, CSCs require more energy than other normal cells due to their high growth and proliferation. Although the glycolytic process provides less energy, CSCs prefer it since it is shorter (Dando et al., 2015). Lactate produced by the glycolytic pathway has the ability to influence CSC function and is involved in processes including metastasis, angiogenesis, and differentiation (Tamada et al., 2012). If the cell is in a state of hyperoxia (low oxygen), it enters the oxidative pathway, where pyruvate created from glucose enters the mitochondria. Then proceeds via the Krebs cycle and OXPHOS pathway to make energy. According to researches, the first alteration in CSC metabolism is a shift from aerobic to anaerobic sugar metabolism, in which oncogenes such as Akt 1 and C-Myc can regulate the glycolytic pathway by acting on the Warburg effect (Dando et al., 2015).

#### Metabolisms Related to Mitochondria

Almost every cell activity relies on the hydrolysis of energy-rich compounds such as ATP. Hereupon, the continuous production and replenishment of such energetic compounds are prioritized by the cells (Dunn and Grider, 2020). Mitochondria are one of the major organelles of cells in which contributes significantly in energy transduction by producing energy-carrying molecules. The mitochondria play a key role by activating the OXPHOS, tricarboxylic acid cycle (TCA), and fatty acid oxidation (FAO) in the cell. Additionally, biosynthetic precursors production, innate immune activation, modulation of the reactive oxygen species (ROS), control of calcium homeostasis, and trigger to apoptotic process are also some of the major activities of mitochondria within a cell (Zong et al., 2016). Owing to the mitochondria biosynthetic and bioenergetics activities, compelling evidence suggests that it also have a crucial impact on CSCs function (De Francesco et al., 2018). The difference in the amount of energy required for cancer stem cells compare to other cells can lead to differences in the quantity of mitochondrial function in them. Studies show that mitochondrial function can be affected by the type of tumor heterogeneity. Evidence also points that epigenetic and micro environmental features are among the factors that can result in altered mitochondrial function in CSCs (García-Heredia and Carnero, 2020). Investigations at the cellular and molecular level imply that changes leading to the production of cancer stem cells can increase the mitochondrial mass (Shin and Cheong, 2019) and membrane potential which are a reflection of electrical and biochemical alterations in CSCs mitochondria (Zhang et al., 2015). Furthermore, changes in mitochondrial DNA (mtDNA) can also affect the expression of some nuclear genes during the retrograde signaling that ultimately lead to inducing EMT process and producing CSCs (Guha et al., 2014). Reciprocally, many mitochondrial proteins are encoded by nuclear DNA (nDNA). Accordingly, mutations or changes in nDNA may eventually lead to altered mitochondrial activity in CSCs (Guerra et al., 2017). In addition to the interaction between mitochondria and the nucleus, disruption of some signaling pathways can affect the role of mitochondria in tumorigenesis. For instance, one of the major functions of PI3K/AKT/mTOR pathway is the regulation of pre-apoptotic proteins such as B-cell lymphoma 2 Associated X, Apoptosis Regulator (BAX) in relation to mitochondria which ultimately leads to apoptosis through this organelle. However, overexpression of apoptosis inhibitor genes in CSCs causes abnormal activation of the mentioned signaling pathway, which can lead to cancer cell proliferation, survival, and drug resistance of cancer cells (Frasson et al., 2015; Liu et al., 2020). Whereas the study of mitochondrial role in relation to other parts of cell on a large scale can be challenging, it should be narrow down the study to the major functions of mitochondria. Therefore, to promote research in the assessment of CSCs, such part particularly focuses on tricarboxylic acid cycle (TCA) and electron transport-linked phosphorylation process, synthesis and degradation of lipids, reactive oxygen species (ROS) generation system, and alternative metabolic pathways such as amino acid metabolism in CSCs.

##### Tricarboxylic Acid Cycle (TCA) and Electron Transport-Linked Phosphorylation Process

Unlike normal cells, CSCs require metabolic adaptation in order to supply fuel and materials for tumorigenesis purposes. Both TCA and OXPHOS which occur alternately following aerobic glycolysis, play an important role in the development of CSCs features. For instance, a reduction in the amount of TCA enzymes can be seen in some CSCs. Additionally, the TCA cycle is associated with different processes such as FAO, glutamine metabolism, and so on. Hence, the TCA cycle can play a key role in the development of stemness capabilities in CSCs under the influence of other metabolites (Yadav et al., 2020). Along with TCA, OXPHOS has an important role in tumorigenesis. As already mentioned, glycolysis is the preferred energy production process compared to the OXPHOS in many CSCs. Although mitochondrial-related bioenergetics processes can produce higher rates of energy-rich compounds, the glycolysis pathway can provide the factors needed for the growth and proliferation of CSCs more timely and rapidly. However, CSCs in some types of cancers such as leukemia, ovarian, glioblastoma, breast, lung, and pancreatic ductal adenocarcinoma (PDAC) do not comply with this rule and prefer the OXPHOS pathway rather than glycolysis (Peixoto and Lima, 2018; Snyder et al., 2018). OXPHOS-dependent CSCs can acquire their needed energy from the uptake and chemical changes on some metabolites such as pyruvate, lactate, ketone bodies, and some amino acids. However, extracellular uptake is not the only way to get the nutrients needed by OXPHOS-dependent CSCs functions (Gentric et al., 2017; Jagust et al., 2019a). They can also supply the required nutrients through metabolic symbiosis with glycolysis to perform their bioenergetics and biosynthetic processes. Interestingly, the restriction of nutrient levels in the surrounding microenvironment of OXPHOS-dependent CSCs has not a huge impact on cell functions. Because in specific tumor microenvironments, they can counteract this limitation with their selective advantages. Therefore, this strategy can make a significant contribution to CSCs survival (Krstic et al., 2017; Zhu et al., 2020). Since mitochondrial-related processes have important effects on the energy and materials supplying of CSCs to grow and develop tumors, targeting different components of these processes can be an efficient approach in the treatment of various types of cancers (Jagust et al., 2019b).

##### Synthesis and Degradation of Lipids

Lipid, as one of the cell membrane`s basic constitutive elements, is necessary for different cell activities, such as signaling conduction, energy production, etc. Sterols, monoglycerides, diacylglycerides, triglycerides, phospholipids, and glycolipids are different components of lipid structure. additionally, most of the lipids originated from fatty acids (Snaebjornsson et al., 2020; Visweswaran et al., 2020). Furthermore, lipid droplets (LDs) act as a lipid storage and in comparison with normal cells, cancer cells have more LDs. Regarding the metabolism of lipid, CSCs have been affected by this kind of metabolism through different strategies such as CSCs maintaining, complying energy desire of CSCs (Visweswaran et al., 2020), increasing CSCs numbers (Mancini et al., 2018), and protecting CSCs from chemotherapeutic agents-induced peroxidation (Begicevic et al., 2019). Moreover, NANOG, sterol regulatory element-binding transcription factor 1(SREBP1), MYC, stearoyl-CoA desaturase (SCD), fatty acid synthase (FASN), ACVL3, CD36, carnitine palmitoyltransferase 1 (CPT1A), and carnitine palmitoyltransferase 1B (CPT1B) are some main modulators for this metabolism. In this respect, there are some alterations in lipid metabolism which lead to different outcomes, such as the effectiveness on the capability of self-renewal, invasion, metastasis, and drug resistance (Giacomini et al., 2020). On the other hand, CSC biomass production, stimulation of the Wnt/-catenin, and Hippo/YAP signaling pathways are some of the other effects that have been linked to CSC activity and cancer progression (Chae and Kim, 2018; Yi et al., 2018; Jagust et al., 2019b). In this context, the altered lipid metabolism can also have some therapeutic effects in the field of CSCs by the CSCs blockage and lessen CSCs chemoresistance ultimately, lipid metabolism contains different signaling pathways that conserve undifferentiating status and the survival of CSCs. Some of these signaling pathways are Notch signaling, Hippo cascades, Hedgehog (Hh) signaling, and Wnt signaling (Giacomini et al., 2020).

##### Reactive Oxygen Species Generation System

In addition to energy production processes, other pathways can play vital roles in multiple aspects of the generation and maintenance of the CSCs function. ROS production is one of these pathways which contribute to cancer recurrence, CSCs metastasis, and resistance to conventional therapies. Generation of ROS can be a consequence of electron transferring through mitochondrial membrane. In addition, enzymes in some other organelles and even immune reactions can play a role in the production of these oxygen species. Studies have also shown that chemotherapy and radiotherapy can eventually lead to increased ROS within cells (Liou and Storz, 2010; Zhou et al., 2014). In general, the antioxidant system acts as a defense barrier against increasing ROS. Maintaining a balance between the amount of antioxidants and ROS can play an important role in cell stability and homeostasis. If this balance is upset and the ROS level increases, cellular stress and eventually cell death occurs (Poljsak et al., 2013; Kurutas, 2016). In contrast, in the case of CSCs, the expression of antioxidants is much higher than in ROS production and keeps the ROS levels low (Shi et al., 2012). Hence, it can promote self-renewal, survival, and resistance to anti-cancer treatments. According to the stated argumentation, ROS can be an appropriate objective for discovering targeted therapies to fight against cancer. For instance, using approaches to increase ROS levels or disruption of antioxidant systems within CSCs can lead to cell aging and apoptosis. Therefore, an effective step can be taken to treat various types of cancer by extensively and accurately examining of ROS modulation in CSCs (Zhou et al., 2014; Ding et al., 2015).

##### Amino Acid Metabolism as an Alternative Metabolic Pathway

CSCs are flexible cells that rely on alternative fuels such as the amino acid glutamine to maximize their growth and proliferation under different environmental conditions (De Francesco et al., 2018). In glucose deficiency, the growth and survival of CSCs are highly dependent on glutamine, which enters the cell through its specific vectors during the path of glutaminolysis and is converted to glutamate by the enzyme mitochondrial glutaminase, thus entering the Krebs cycle (Deshmukh et al., 2016). Glutamine, as a source of nitrogen, plays an important role in mediating metabolites, which eventually synthesize various substances, including protein, lipids, and nucleotide acids (Deshmukh et al., 2016). CSCs of various tumors, including the pancreas, pancreas, ovaries, and lungs, are glutamine-dependent (Deshmukh et al., 2016). The pentose phosphate (PPP) pathway is also used as an alternative pathway for fuel generation in CSCs. PPP is performed in two forms: reversible (non-oxidative) and irreversible (oxidative) (Giacomini et al., 2020), which is an alternative pathway for glucose metabolism during the irreversible pathway of PPP. In this pathway, glucose 6-phosphate (G6P) is converted to ribose 5-phosphate in several steps with the production of nicotinamide adenine dinucleotide phosphate (NADPH), and finally essential nucleotides are synthesized by forming ribose groups (Riganti et al., 2012; Polat et al., 2021). However, in reversible PPP, ribose 5-phosphate is converted to glyceride aldehyde 3 phosphate in a series of reversible reactions and is eventually used for glycolysis (Polat et al., 2021). Ketone bodies (acetone, acetate, 3-hydroxybutyrate) are among the high-energy fuels used by CSCs to grow and propagate metastases (Jagust et al., 2019b). When there is not enough glucose in CSCs, ketone bodies are released into the blood and converted directly to Acetyl-CoA by the two enzymes OXCT1 and ACAT1. Then acetyl-CoA enters the citric acid (CAC) cycle and produces more ATP in the cell (Ozsvari et al., 2017). In addition to glutamine, lysine is another amino acid that CSCs use to make fuel, as well as TICs, which contain many enzymes; They perform the process of lysine catabolism (Jagust et al., 2019b). As a result of the lysine pathway, glutamate is synthesized and cysteine uptake is increased in CSCs (Peixoto and Lima, 2018).

### The Chemoresistance of Cancer Stem Cells

Chemoresistance is defined as a pivotal factor of defeated chemotherapy treatment in various cancers. This factor relapses affected agents of chemotherapy such as cell death and tumor bulk`s size decrement. In this respect, CSCs considerably execute the role of referred relapsing and also it has the capability of showing resistance against chemotherapeutics by its insensitivity (Abdullah and Chow, 2013; Zhao, 2016). Chemoresistance of CSCs leads to a high risk of metastases, less survival speed (Nunes et al., 2018), and the permanence of CSCs (Chuthapisith et al., 2010). Furthermore, a comparison between normal cancer cells and CSCs revealed that, CSCs intrinsically have a higher amount of chemotherapy resistance than normal cancer cells (Thomas et al., 2014). Many factors are involved in CSCs resistance occurrence, which some of them are as detailed below:

• *Tumor microenvironment (TME):* One of the factors involved in the regulation of stemness characteristics and chemoresistance of CSCs, is the autocrine and paracrine interactions of CSCs with the components of their surrounding environment, which is referred to as the TME. In recent years, the key role of TME and its components including extracellular matrix, immune cells, endothelial cells, cancer-associated adipocytes (CAAs), and cancer-associated fibroblasts (CAFs) in the onset, metastasis, recurrence, and drug resistance of cancer have been investigated. The results of these studies show that targeting the TME can be an effective approach in the treatment of cancer (Gaggianesi et al., 2021).
• *Epigenetics*: Another major factor in the chemoresistance of CSCs is the role of mechanisms followed epigenetic alterations. Studies reveal that epigenetic processes such as DNA methylation, nucleosome remodeling, histone modification, and non-coding RNAs changes are generally associated with the development of normal stem cell characteristics. However, disruption in the normal function of epigenetic factors lead to the development of tumorigenic properties in CSCs (Toh et al., 2017).
• *Epithelial Mesenchymal Transition (EMT)*: EMT is a biological phenomenon during processes such as embryonic development, wound healing, and tissue regeneration. However, in the case of cancer, EMT can suppress epithelial features and convert the cell into the mesenchymal state through signaling pathways such as Wnt, Notch, and Hedgehog, which can lead to the development of tumor features (Singh and Settleman, 2010).
• *Multidrug resistance (MDR)*: High levels of MDR is another main factor involved in the chemoresistance of CSCs that occurs after applying long-term or high-dose treatment for cancer patients. Generally, two mechanisms can be considered for the effect of MDR on CSCs: 1) Preventing the drug from reaching an effective concentration: studies imply that the function of efflux pumps such as P-glycoprotein (P-gp) encoded by ABCB1, transporters, and enzymes such as cytochrome P450 and glutathione S-transferase play significant roles in mediating drug resistance. 2) Drug detoxification: based on studies, it has been realized that avoiding apoptosis and activating DNA repair mechanisms are of fundamental importance to induce continuous growth and proliferation of CSCs. (Cho and Kim, 2020).
• *The quiescent state*: Quiescence or dormancy is a survival strategy for CSCs. In the quiescent state, cell division stops for a while, and cells live with minimal metabolic activity, but still retain the ability to reactivate the cell cycle (Chen et al., 2021). In this state, both intrinsic (e.g., p53 signaling, reactive oxygen species, hypoxia inducible factor-1a, nuclear factor of activated T cells c1, and negative regulators of mTOR) and non-intrinsic factors (e.g., Tie2/angiopoietin-1, TGF-b and bone morphogenic proteins, thrombopoietin, N-cadherin and integrins, osteopontin, and Wnt/b-catenin signaling) are involved (Li and Bhatia, 2011). According to studies, TME and epigenetic mechanisms have a major contribution to the maintenance of the quiescentstate of CSCs as well as evade immune surveillance and destruction, and tumor relapse. Therefore, the presence and persistence of the quiescence or dormancy state in CSCs can lead to the survival of CSCs and cell resistance to treatments such as chemotherapy (Chen et al., 2021).
• *Self-renewal*: Self-renewal is one of the noted hallmarks of CSCs that results from a malfunction of self-renewal pathways (SRPs). Studies indicate that Hh, Wnt, Notch, and B-cell-specific Moloney murine leukemia virus integration site 1 (BMI1) pathways have a crucial role in inducing the self-renewal in CSCs. In recent years, the targeting of SRPs has attracted attention as an efficient therapeutic approach in cancer treatment to reduce cancer recurrence and chemotherapy resistance possibility (Borah et al., 2015).

It should also be noted that according to metabolic studies, cancer cells that have undergone chemoresistance are metabolically altered and adapted. For example, processes such as fatty acid oxidation, glutaminolysis activation, glycolysis activation, lactate production, adaptive mitochondrial reprogramming, ornithine decarboxylase, and polyamine production, and PPP and NADPH production can be observed in chemoresistance cancer cells (Chen et al., 2020).

## Molecular Docking Study; A Therapeutic Approach for Anti-Cancer Drug Designing

Drug Discovery is considered as a multi-process platform in which a specific chemical compound with desired biological activity on the drug target can be selected and eventually enter the drug development as a candidate drug. In this platform, both chemical compounds and biological targets are evaluated from different aspects by using various approaches. Since both drug discovery and development are time-consuming and cost-intensive programs, they pose many challenges for researchers in drug designing and discovering for various diseases such as different types of cancer. Therefore, the use of new technologies can pave the way discovering new drugs with high therapeutic potential and take a big step towards disease treatment. Compound screening assays are one of mentioned methods that can help with hit identification, validation, lead generation, and optimization processes, as well as evaluating the compounds’ effects on the therapeutic target. With the advancement of technology and the integration of computational science with biological and pharmaceutical studies, approaches such as virtual screening are widely applied in drug designing and discovering programs (Reddy et al., 2007; Hughes et al., 2011; Mohs and Greig, 2017; Cui et al., 2020). In this context, virtual screening aims to evaluate and filter a limited number of suitable chemical compounds from large libraries of small molecules by using mathematical calculations. Structure-based virtual screening (SBVS) is one of the virtual screening methods which attempts to model and analyze the efficient biological binding-conformation between a ligand and a target molecule by using the molecular docking technique (Liao et al., 2013). Molecular docking is one of the cutting-edge computational drug designing technologies in which the most effective and stable state form of the ligand-receptor complex can be predicted (Morris and Lim-Wilby, 2008). The determination of the three-dimensional structure of the target and ligand molecules is at the top of the entire process priority list. Therefore, some techniques such as x-ray crystallography, nuclear magnetic resonance (NMR) spectroscopy, cryo-electron microscopy (cryo-EM), and homology modeling are not only useful in determining molecular structure, but also as complementary tools in drug development (Allen and Stokes, 2013; Kershaw et al., 2013; Sturlese et al., 2015; Lohning et al., 2017). Molecular docking includes searching algorithms and scoring function as two fundamental aspects of docking programs. Searching algorithms can be defined as a process that can lead to exploring the predominant and effective matching docking modes of ligand to the molecular target among the myriad configurations. Because a large number of binding modes are actually found between a ligand and a biological target molecule, searching algorithms not only can consider the optimum possible orientations of the ligand with the target but also can be an economical and time-saving solution in the docking process (Meng et al., 2011; Salmaso and Moro, 2018). Molecular dynamics, distance geometry methods, point complementary methods, fragment-based methods, Mote Carlo methods, genetic algorithms, systematic searches, and incremental construction, are some of the examples of search algorithms that can be used in modeling and evaluating the binding form of a ligand molecule to the objective receptor**.**

After the algorithm searching, it is time for the scoring function to step into the docking arena to find the good pose between the ligand and the target molecule. Scoring function refers to a process in which putative docking modes are ranked by evaluating their binding affinity and lowest binding energy to achieve top-ranked poses between a ligand and a target molecule. Force field function, Empirical scoring functions, knowledge based scoring functions, knowledge-based potentials, machine learning based scoring functions, comparative assessment of scoring functions, physics-based methods, and descriptor-based scoring functions are some of the examples of scoring function classifications in molecular docking (Madhavilatha and Babu, 2019; Sethi et al., 2019).

**FIGURE 1:**
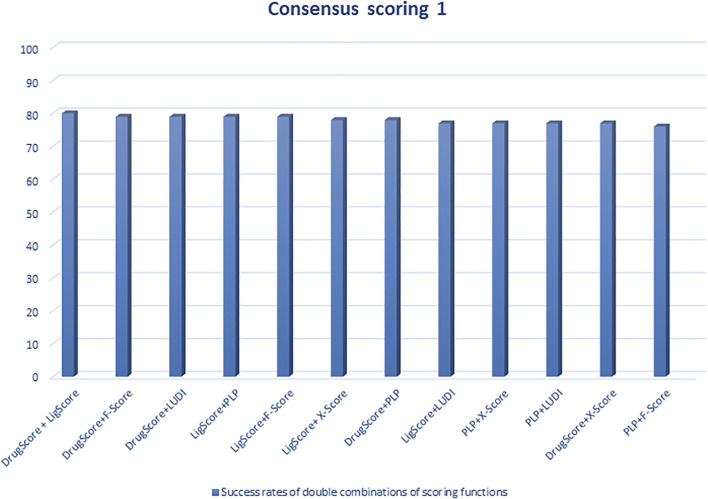
Success rates of double combinations of the six relatively successful scoring functions in consensus scoring. All numbers are in percent (Wang et al., 2003).

**FIGURE 2:**
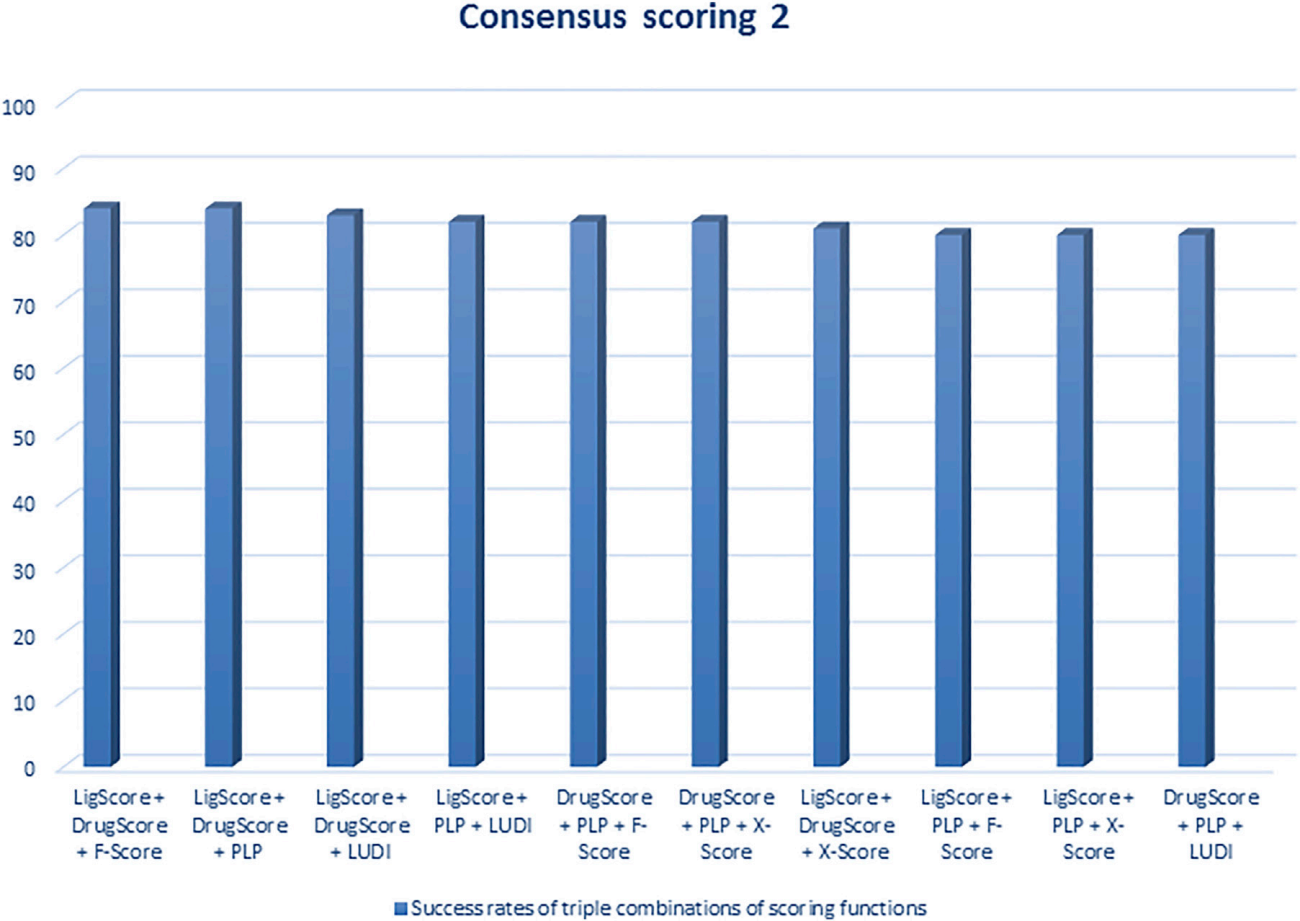
Success rates of triple combinations of the six relatively successful scoring functions in consensus scoring. All numbers are in percent (R. Wang et al., 2003).

Regarding scoring function, the study of Wang et al. (2003) is one of the best examples of meticulous studies of popular scoring functions in molecular docking. In this study, the authors compared 11 popular scoring functions, including four scoring functions of the LigFit module in Cerius2 (LigScore, PLP, PMF, and LUDI), four scoring functions of the CScore module in SYBYL (FScore, G-Score, D-Score, and ChemScore), the scoring function of the AutoDock program, and two stand-alone scoring functions (DrugScore and X-Score) by performing them on 100 protein−ligand complexes to scrutinize their performance and sift the most effective and efficient methods among them. In this regard, after generating a set of docked conformations for each ligand by Autodac software, each 11 scoring function was tested and implemented on the maintained set and significant results were obtained. In this study, the authors used root-mean-square deviation ≤2.0 Å as a criterion for examining those scoring functions. Based on the analysis of the mentioned criterion, it was concluded that six scoring functions, including PLP, F-Score, LigScore, DrugScore, LUDI, and X-Score achieved high success rates (about 66–76%). In addition, the study implied that the combination of some of those scoring functions and generating the consensus scoring scheme can also increase the success rate (about 80%) (Figures 1, 2). In addition to success rates, the authors examined the correlation between 100 complexes’ binding scores and experimentally determined protein-ligand binding affinities. As a result of this experiment, X-Score, PLP, DrugScore, and G-Score could represent correlation coefficients of more than 0.50, which demonstrate superiority over other scoring functions. Since the best scoring function should perform excellently in both docking and scoring, the three scoring functions, including X-Score, DrugScore, and PLP, can be considered the top scoring functions in molecular docking, according to the study by Wang et al. (Wang et al., 2003).

## Targeting Cancer Stem Cells Metabolic Process by Molecular Docking

Molecular docking is a substantial method for estimating the interaction between macromolecules such as protein and small molecules such as ligand. On the other hand, molecular docking is also capable of analyzing the molecular kinds of behavioral variability for those molecules which are located at the binding site of a targeted protein. Also, molecular docking is a computational approach and some docking programs are required to carry out its many functions. Some of the most considerable docking programs are Gold, Fred, and Flex. Moreover, they are useful in the prevision of protein and ligand`s binding conjunction (Kumar et al., 2013; Pagadala et al., 2017). In this respect, molecular docking is applied in different CSCs-related pathways including metabolic pathways and signaling pathways. Shedding light on metabolic pathways, the activity modification which can be applied by cancer cells, result in the production of metabolic precursors which leads to cancer cells anabolic and energetic requirement fulfilling. Furthermore, different metabolic pathways take a part in tumor progression and malignant tumor alterations. Accordingly, metabolic reprogramming is considered as one of the cancer insignias (Jagust et al., 2019b).

Herein, there will be a few examples in the context of some molecular docking usages in metabolic pathways such as 1) mitoketoscins application in targeting metabolic tumor promoters (OXCT1 and ACAT1) in both ketone re-utilization and mitochondrial function (Ozsvari et al., 2017). 2) Survivin protein interaction with andrographolide, which can lead to having an influence on human breast CSCs apoptosis (Wanandi et al., 2020). 3) S-phase kinase-associated protein-2 (Skp2) inhibition process by compound #25 which can result in CSCs survival suppression (Chan et al., 2013).

The other molecular docking-affected pathway in CSCs is the signaling pathway. This pathway is useful for targeted CSCs therapies expansion (Koury et al., 2017), embryonic evolvement, maintaining CSCs, etc. (Karamboulas and Ailles, 2013). Some molecular inhibitor agents of Wnt, Notch, Hh, and some other signaling pathways are implying as one of the important effects of molecular docking process on signaling pathways (Yang Y et al., 2020).

Modulating some target proteins is a striking aspect of molecular docking which has done by natural products. Natural products are able to be considered on the ground of multi-targeting drugs. As such, alkaloids are one of the natural products that have the strength to act as an anticancer lead molecule in the molecular docking process of CSCs. In this regard, Jaitak et al. provided an in-depth analysis of multitargeting drugs as an effective strategy to fight against CSCs and prevent disease recurrence. In this study, the authors examined the effect of some alkaloids that have anticancer potentiality by focusing on the Hh pathway in cancer stem cells. After selection and preparation of target ligands and proteins, Grid parameter selection and validation, implementation of glide docking module of Schrö6; dinger Maestro 9.6 suite, and determination of ADME profile for the studied alkanoid ligands, significant results were discovered. For instance, according to the findings of this study, emetine, and cortistatin, were able to target CSCs maintenance feature by binding to sonic Hh, smoothened (Smo) and, gli protein. Therefore, these two drugs can be applied as multi-targeting drugs in a combination with cancer chemotherapy compounds. Moreover, solamargine alkaloid could also have a good effect on gli protein and sonic hedgehog due to its pharmacophores. Furthermore, both solasonine and tylophorine modulated the Hh pathway and exert anticancer effects on CSCs by affecting only gli proteins. However, unlike other drugs, solamargine and solasonine need to improve the properties of ADME features (Jaitak, 2016).

In addition to alkaloids, the targeting of overexpressed CD44 surface marker in triple-negative breast cancer (TNBC) tissues can have an anticancer effect on CSCs. Regarding targeting CD44 surface markers, Yang et al. determined the positive role of drug carriers including Gambogic acid (GA)-loaded, zirconium-89 (89Zr)-labeled, chitosan (CS)-decorated multifunctional liposomes (MLPs) on TNBC CSCs by designing two *in vitro* and *in vivo* experiments. In this study, researchers examined 3D mammospheres and TNBC tissues of 32 women who were diagnosed with TNBC and found that the CD44 surface marker was overexpressed in the disease. Therefore, in this study, 89Zr@CS-MLPs were constructed and predicted how the drug carriers interact with CD44 surface markers in TNBC by applying molecular docking and dynamics simulations methods. The results obtained from the *in vitro* (examination on tumor cell lines) and *in vivo* (examination on mice) experiments were implied that 89Zr@CS-MLPs has a great potentiality for TNBC-targeted therapy as a drug carrier. Additionally, since Zr has a long half-life, it can also be used as an ideal radiolabel for positron emission tomography (PET) imaging of cancer. Moreover, 89Zr@CS-GA-MLPs have a high ability to target CSCs *in vivo* (Yang R et al., 2020).

**FIGURE 3:**
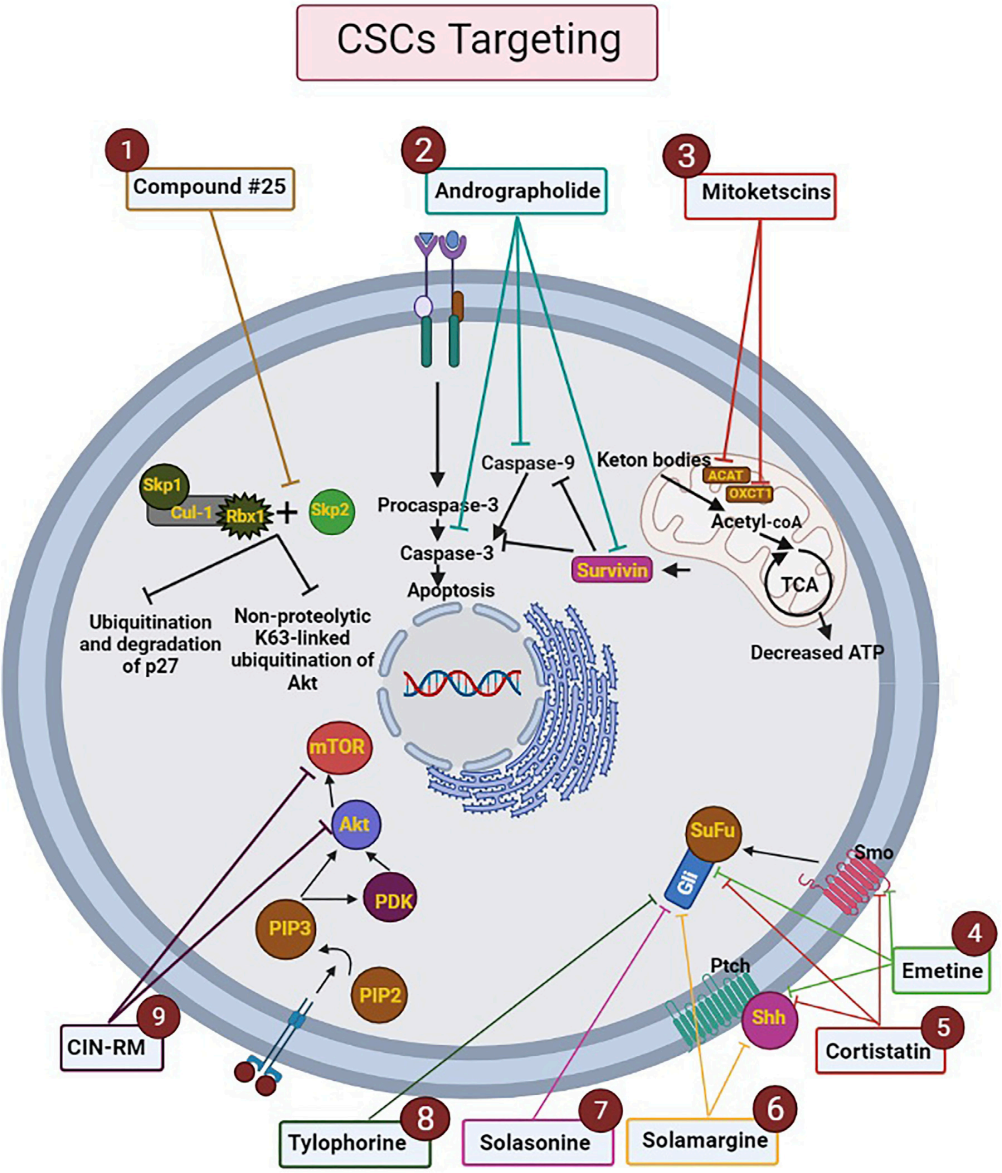
Mechanism of action of drugs analyzed by molecular docking on the metabolic processes of CSCs. The ligands and targets have been investigated by molecular docking. Nine effective drugs, including compound #25, andrographolide, mitoketoscins, emetine, cortistatin, solamargine, solasonine, tylophorine, and CIN-RM are known to affect the biological processes and signaling pathways of CSCs. 1) Compound #25 prevents the assembly of the Skp2-SCF complex by binding to Skp2. Hence, it inhibits two pathways including non-proteolytic K63-linked ubiquitination of Akt and ubiquitination and degradation of p27, which ultimately inhibit the development of tumor features. 2) Andrographolide increases intrinsic apoptosis in CSCs (especially in breast cancer) by inhibiting survivin, caspase-9, and caspase-3.3) Mitoketoscins stop the recycling of ketone bodies into Acetyl-CoA by inhibiting two proteins, including OXCT1 and ACAT1. Hence ATP production is stopped and oxidative mitochondrial metabolism in CSCs is inhibited. 4) Emetine and 5) Cortistatin, can target CSCs by binding to sonic Hh, Smo and, gli protein. 6) Solamargine can affect sonic hedgehog and gli proteins by its pharmacophores. 7) Solasonine and 8) Tylophorine modulate the Hh pathway by affecting gli proteins. 9) CIN-RM can lead to upstream inhibition of the Akt pathway and reduction of CSCs markers, which decrease the expression level of transcription factors involved in self-renewal, such as c-Myc, Nanog, Oct4, and Sox2. CIN-RM can inhibit mTOR pathway. Abbreviations: ATP, Adenosine triphosphate; CIN-RM, Hydroquinone 5-O-cinnamoyl ester of renieramycin M; CSCs, Cancer stem cells; Hh, Hedgehog; mTOR, Mammalian target of rapamycin; Smo, smoothened (Chan et al., 2013; Hongwiangchan et al., 2021; Jaitak, 2016; Liu et al., 2014; Madhunapantula et al., 2011; Ozsvari et al., 2017; Wanandi et al., 2020).

In 2021, Hongwiangchan et al. demonstrated that hydroquinone 5-O-cinnamoyl ester of renieramycin M (CIN-RM) can be recognized as a fundamental approach in targeting lung CSCs which has been confirmed by molecular docking computational analysis. The effect of CIN-RM is based on the reduction of CSCs markers and upstream inhibition of the AKT pathway. As a result of inhibition of Akt, the expression level of transcription factors involved in self-renewal, such as c-Myc, Nanog, Oct4, and Sox2, are decreased. It is, therefore, CSCs are suppressed and tumor growth can be inhibited. To a lesser extent, CIN-RM also induces its inhibitory effect on the mTOR pathway, but the inhibitory effect of CIN-RM on the protein kinase C (PKC) signal pathway was not significant. Another important result obtained in this study is that CIN-RM even has an effect on inactivating the AKT pathway related to c-Myc regulation of lung non-stem cancer cells, which can be a promising therapeutic approach in cancer treatment (Figure 3) (Hongwiangchan et al., 2021).

## Conclusion and Future Perspective

A comprehensive analyzing of CSCs, including signaling pathways and metabolic activities, as well as recognizing their distinctions with normal cells, is an essential technique in cancer targeted treatment (Bjerkvig et al., 2005; Shyh-Chang and Ng, 2017). Major obstacles to molecular binding as a targeted therapeutic technique include receptor flexibility, ligand flexibility, and drug resistance, all of which have contributed to cancer therapy failure (Meng et al., 2011). Computational methods or computer tools, which are a form of artificial intelligence (AI), have recently proven to be a useful approach in a variety of domains, including structure prediction, molecular bond modeling, and junction prediction (Menke et al., 2021). The mentioned methods are divided into two groups: classical and machine learning (ML), of which ML is widely used in molecular binding. Computational methods play a key role in molecular binding for drug design and discovery, and results can be analyzed cheaper and often faster than other conventional methods (Torres et al., 2019). Eventually, with the advancement of science and the identification of various computational methods, computing software and hardware are still being updated, and researchers are looking for the most accurate way to target CSCs for cancer treatment (Phillips et al., 2018).

## Glossary

IARC: international agency for research on cancer
WHO: world health organization
CSCs: cancer stem cells
MRPs: multidrug resistance proteins
EMT: epithelial-to-mesenchymal transition
OXPHOS: oxidative phosphorylation
hESCs: human embryonic stem cells
NF-κB: nuclear factor-κB
JAK-STAT: janus kinase/signal transducers and activators of transcription
PI3K/AKT/mTOR: phosphoinositide 3-kinase/AKT/mammalian target of rapamycin
TGF: transforming growth factor
PPAR: peroxisome proliferator-activated receptor
ATP: adenosine triphosphate
TCA: tricarboxylic acid cycle
FAO: fatty acid oxidation
ROS: reactive oxygen species
Skp2: S-phase kinase-associated protein-2
mtDNA: mitochondrial DNA
nDNA: nuclear DNA
BAX: B-cell lymphoma 2 associated X, apoptosis regulator
PDAC: pancreatic ductal adenocarcinoma
LDs: lipid droplets
SREBP1: sterol regulatory element-binding transcription factor 1
SCD: stearoyl-CoA desaturase
FASN: fatty acid synthase
CPT: carnitine palmitoyltransferase
Hh: hedgehog
PPP: pentose phosphate pathway
G6P: glucose 6-phosphate
NADPH: nicotinamide adenine dinucleotide phosphate
CAC: citric acid cycle
NMR: nuclear magnetic resonance
Cryo-EM: cryo-electron microscopy
TICs: tumor-initiating cells
SBVS: structure-based virtual screening
EM: electron microscopy
TNBC: triple-negative breast cancer
Smo: smoothened
GA: gambogic acid
CS: chitosan
MLPs: multifunctional liposomes
CIN-RM: hydroquinone 5-O-cinnamoyl ester of renieramycin M
PET: positron emission tomography
PKC: protein kinase C
AI: artificial intelligence
