# Corrigendum: Molecular Docking as a Therapeutic Approach for Targeting Cancer Stem Cell Metabolic Processes

**DOI:** 10.3389/fphar.2022.892656

**Published:** 2022-05-02

**Authors:** Babak Arjmand, Shayesteh Kokabi Hamidpour, Sepideh Alavi-Moghadam, Hanieh Yavari, Ainaz Shahbazbadr, Mostafa Rezaei-Tavirani, Kambiz Gilany, Bagher Larijani

**Affiliations:** ^1^ Cell Therapy and Regenerative Medicine Research Center, Endocrinology and Metabolism Molecular-Cellular Sciences Institute, Tehran University of Medical Sciences, Tehran, Iran; ^2^ Proteomics Research Center, Shahid Beheshti University of Medical Sciences, Tehran, Iran; ^3^ Integrative Oncology Department, Breast Cancer Research Center, Motamed Cancer Institute, ACECR, Tehran, Iran; ^4^ Reproductive Immunology Research Center, Avicenna Research Institute, ACECR, Tehran, Iran; ^5^ Endocrinology and Metabolism Research Center, Endocrinology and Metabolism Clinical Sciences Institute, Tehran University of Medical Sciences, Tehran, Iran

**Keywords:** cancer, cancer stem cells, drug designing, metabolic processes, molecular docking

In the published article, there was an error in affiliation 3, 4, 5. Instead of “3 Reproductive Immunology Research Center, Avicenna Research Institute (ARI), Tehran, Iran, 4 Department of Biomedical Sciences, Institute of Tropical Medicine Antwerp, Antwerp, Belgium, 5 Breast Cancer Research Center, Motamed Cancer Institute, Tehran, Iran”, it should be “3 Integrative Oncology Department, Breast Cancer Research Center, Motamed Cancer Institute, ACECR, Tehran, Iran, 4 Reproductive Immunology Research Center, Avicenna Research Institute, ACECR, Tehran, Iran, 5 Endocrinology and Metabolism Research Center, Endocrinology and Metabolism Clinical Sciences Institute, Tehran University of Medical Sciences, Tehran, Iran”.

The authors apologize for this error and state that this does not change the scientific conclusions of the article in any way. The original article has been updated.

